# The Royal Photographic Society's Exhibition

**Published:** 1913-09-13

**Authors:** 


### THE ROYAL PHOTOGRAPHIC SOCIETY'S EXHIBITION.

### Examples of Radiographs, Photomicrography, and Colour Work.

The fifty-eighth annual exhibition of the Royal Photographic Society is now being held at the Gallery of the Royal Society of British Artists, Suffolk Street, Haymarket, and an inspection will well repay the institutional visitor. He will see a representative collection, pictorial and scientific, produced by the best-known workers. It is of course the technical sections which will more especially attract our readers, there being much of interest in the way of medical and biological subjects, with some admirable examples of *x*-ray work. A few remarks, however, on the pictorial collection may not be out of place, embodying as it does the most modern processes by recognised experts. The standard of work shown is at least equal to that of previous years, and there is a welcome absence of the extreme " fuzzy " productions which were formerly so much in vogue.

Foremost among the art contributions are the well-known works of Robert Demachy, Hoppé, Puyo, Duhrkoop, Frank Read, and others. "Danseuse," by Puyo, and the exhibits of Demachy are some of the most noteworthy. All the exhibits in this section are of much interest by reason of their artistic merit and subdued effects, while in it there are a number of prints in natural colours produced by the photogravure, bromide, and oil processes. Section II. is devoted to colour transparencies, the technique and correct colour values of which leave little to be desired, and here we notice a marked advance on the work of previous years. Three medals have been awarded to the exhibits in this section and these should on no account be missed.

### Tracking Adulteration and Colour Work.

Scientific and technical exhibits in natural history, radiography, microscopy, colour prints, lantern and stereoscopic slides are included in Section III., and it is here that the interest of our readers will be more particularly alert. The subject of "diffusion of matter" is original work by Dr. Stephane Leduc, illustrating the solution of sugar, salt, etc., in different fluids. Of much scientific interest is the exhibit of original negatives of the spectra of metals by Lieut.-Col. Gifford; photography on copperplates, by G. Reboul; and the medalled work of Ernest Marriage. The latter represents methods of microscopical and micro-chemical analysis of colloids—jams in particular—and is of economic value. Here we see specimens of adulterated jams examined by polarized light, strawberry, raspberry, and gooseberry, with colloidal tests of the fruit jellies. The presence of such adulterants as starch, inferior fruit jellies, honey and glucose are, by selective chemical treatment, conclusively demonstrated.

Colour photographs on paper, by Louis Johnson, are worth close inspection, being some of the best results we have seen in this process. A. E. Bawtree shows an original process for the production of colour transparencies, which make a most effective display, the essential principle of which is the dyeing of a fish-glue print with aniline dyes; several distinct points of novelty are here demonstrated. Wratten and Wainwright, the specialists in panchromatic photography, show some fine examples of their work side by side for comparison with prints by the ordinary processes. These will appeal to the photographic expert and are most convincing.

In natural history subjects the exhibition, as in previous years, is especially strong. Botany and flower photography are illustrated, among others, by Dr. G. Rodman, a well-known worker, in the anther of convolvulus; galls caused by aphides, by A. W. Dennis; some very perfect examples of flower photography, by Vincent Young, and beautiful Australian botanical subjects, by W. J. Newman. Bird photography is always a feature at the Royal, and this year is no exception. A medal is awarded to Alfred Taylor for a series of twenty prints of the life-history of the cuckoo from egg to adult. Others comprise the ringed plover, penguin, grey lag goose, tern, vulture, wagtail, etc. Examples of instantaneous studies of animal life are also seen. Those of the alligator and crocodile, by Martin Duncan, are of more than ordinary interest. The entomologist is well catered for. Dr. Drake-Brockman shows photographs of living specimens of the lappet, emperor, and puss moths; the stick insect, by the same worker, is a splendid example of careful photography. Hugh Main exhibits the metamorphoses of the maple-leaf-cutter sawfly.

### Skiagraphs and Radiography.

Radiography is illustrated by Pierre Goby (medallist), showing the value of the soft *x*-rays in insect radiography; such results have not hitherto been obtainable by *x*-rays nor by the use of radium, as regards contrast or clearness of definition. The examples shown are micro-radiographs of small vertebrates, plant anatomy, foraminifera and diatoms. Dr. G. Rodman also shows radiographs of fossils and mollusca. Dr. Bela Alexander exhibits some exquisite plastic skiagraphs of metal objects, wire gauze, portion of human skull and bones of a healthy living hand showing solid appearance of the bones with a surface view. Dr. Gilbert Scott has prints from skiagraphs illustrating the value of instantaneous radiography in the examination of the intestines (such as that favourite instance for showing their appearance immediately after a bismuth meal), gastric ulcer, duodenum, bismuth in stomach, hour-glass stomach, and perforating ulcer an hour after a bismuth meal. Dr. Thurstan Holland has similarly good work in radiographs of stomach, showing the bismuth food spreading between the stomach walls and the hair balls, and also between the latter; others show the various forms of hour-glass constriction following gastric ulcer; the old hour-glass constriction and the common form showing ulcer cavities on lesser curvature. Dr. Robert Knox has a fine series of good work, such as tuberculous knee-joint, loose bodies in knee-joint, pelvis with tube in left ureter, intracapsular fracture of neck of femur, and notably the adult thorax (exposure of 1-85th second); another showing movements of the diaphragm with respiratory movements, and one of enlarged heart, chronic lung disease, and spinal curvature.

In photomicrography those of the corpuscular elements of human blood, by Dr. D. H. Hutchinson, are photographed by 750 to 2,250 diameters and represent good work of this kind. Photographs of the chigoe or jigger flea, by G. Ardaseer, are of interest, as are also those illustrating the histology of the optic nerve of sheep by J. T. Holder. Photomicros of diatoms, by J. M. Offord, complete the series of exhibits in this class.

The exhibition remains open until October 4, and lantern lectures are being given by well-known authorities on various subjects every Tuesday, Thursday, and Saturday, at 8.30 p.m.

